# Genome sequence of the marine alphaproteobacterium *Lentilitoribacter* sp. EG35 isolated from the temperate octocoral *Eunicella gazella*

**DOI:** 10.1128/mra.00872-24

**Published:** 2024-10-24

**Authors:** Tina Keller-Costa, Selene Madureira, Ana S. Fernandes, Lydia Kozma, Jorge M.S. Gonçalves, Cristina Barroso, Conceição Egas, Rodrigo Costa

**Affiliations:** 1 Institute for Bioengineering and Biosciences and Institute for Health and Bioeconomy (i4HB), Instituto Superior Técnico, Universidade de Lisboa, Lisbon, Portugal; 2 Department of Bioengineering, Instituto Superior Técnico, Universidade de Lisboa, Lisbon, Portugal; 3 École Polytechnique Fédérale de Lausanne, Écublens, Switzerland; 4 Centre of Marine Sciences (CCMAR), University of Algarve, Campus de Gambelas, Faro, Portugal; 5 Biocant—Transfer Technology Association, BiocanPark, Cantanhede, Portugal; 6 Center for Neuroscience and Cell Biology (CNC), Rua Larga-Faculdade de Medicina, University of Coimbra, Coimbra, Portugal; California State University San Marcos, San Marcos, California, USA

**Keywords:** bacterial evolution, corals, host-microbe interactions, *Rhizobiaceae*, symbiosis

## Abstract

We report the genome sequence of *Lentilitoribacter* sp. strain EG35 isolated from the octocoral *Eunicella gazella* sampled off the coast of Portugal. We reveal the coding potential for the biosynthesis of polyhydroxyalkanoates — biodegradable polyesters that may serve bioplastics production, diverse homoserine lactone-like communication signals, and four putatively novel natural products.

## ANNOUNCEMENT

The microbial communities of octocorals are unique in taxonomic composition and presumably benefit their host through nutrient provision and cycling, antioxidant production, and chemical defense (1–3). *Lentilitoribacter* (*Rhizobiaceae*, *Hyphomicrobiales*) is a marine alphaproteobacterial genus currently with only one valid species*, Lentilitoribacter donghaensis* (4) and one RefSeq genome (5, 6) available. It has so far been cultured from seawater (4), marine sponges (5), and octocorals (7), yet little is known about its role in association with marine invertebrates.

We report the genome of *Lentilitoribacter* sp. strain EG35 isolated from a healthy *Eunicella gazella* specimen, sampled by SCUBA diving at 18 m depth in the Atlantic off the coast of Portugal (Pedra da Greta: 36.979778, –7.98911) on 21 April 2021. A microbial cell suspension was retrieved from 1 g of coral soft tissue by mortar-and-pestle homogenization in 9 mL sterile Ca2+ and Mg2+-free artificial seawater as described earlier (8). Serial dilutions were plated on Marine Agar (MA) and incubated for 7 days at 24°C. Single colonies were streaked until purity on MA plates, and genomic DNA was extracted from a pure culture, freshly grown in Marine Broth using the Wizard Genomic DNA Purification kit (Promega, USA). Strain EG35 was identified by Sanger sequencing of the 16S rRNA gene amplified from genomic DNA using primers F27 (5′-AGAGTTTGATCMTGGCTCAG-3′) and R1492 (5′-TACGGY TACCTTGTTACGACTT-3′) and the SILVA/SINA (v1.2.12) database for taxonomic classification. The same DNA sample was used for genome sequencing at GENOINSEQ (Biocant, Cantanhede, Portugal). The genome library was prepared with the Nextera XT DNA Library Preparation Kit, and the genome was paired-end sequenced (average read length, 257/258 bp) on an Illumina MiSeq sequencer with the MiSeq reagent Kit v3 (600 cycles). Default parameters were used for all software unless otherwise specified. Raw reads were imported to KBase (9) and quality-checked using FastQC v0.12.1. Low-quality reads were removed using Trimmomatic v.0.36 (10) prior to genome assembly using SPADES v3.15.3 (11). Contigs below 500 bp were removed. Genome completeness and contamination were assessed with CheckM v1.0.18 (12), and genome taxonomy was confirmed with GTDB-Tk v2.3.2 (13). Contigs were annotated using the NCBI Prokaryotic Genome Annotation Pipeline (PGAP v.6.7) (14) and our in-house Melange pipeline (https://github.com/sandragodinhosilva/melange). AntiSMASH v7.0 (15) was used to identify secondary metabolite biosynthetic gene clusters (BGCs).

The general features of the genome are shown in Table 1. Strain EG35 shared 98.39% average nucleotide identity (ANI)—calculated with FastANI v0.1.3 (16)—with *Lentilitoribacter* sp. Alg239-R112 (GCF_900537175.1), its closest relative with a sequenced genome as determined by phylogenomics inference. Strain Alg239-R112 was isolated from the marine sponge *Spongia* sp (5). at the same sampling location where strain EG35 was retrieved.

**TABLE 1:** General features of the *Lentilitoribacter* sp. EG35 genome reported in this study

							Estimate (%) of:		Number of:		Counts of:				Counts of CDs in^*b*^					
Genome size (bp)	GC-content (%)	Genome coverage (x)	Number of contigs	ContigN50 (bp)	Number of reads	Average Read length (bp)	Completeness (%)	Contamination (%)	Coding sequences^a,b^	Genes^*a*^	RNA^*a*^	rRNA^*a*^	tRNA^*a*^	ncRNA^*a*^	COGs^*b*^	Pfams^*b*^	GenBank accession number	SRA accession number	Bioproject accession number	Biosample accession number
4,063,774	44.5	243.4	45	307,952	2×1,714,668	257/258	99.92	0.00	3,935^*a*^ 3,912^*b*^	3,977	42	3	35	4	3,131	7,130	JBFNGQ00000000	SRR29864805	PRJNA1135483	SAMN42483928

^*a*^ Annotation performed by the NCBI Prokaryotic Genome Annotation Pipeline (PGAP): https://www.ncbi.nlm.nih.gov/genome/annotation_prok/

^*b*^ The Melange pipeline (https://github.com/sandragodinhosilva/melange) was used to perform Protein family (Pfam) and Clusters of Orthologous Groups of Proteins (COG)-based annotations of the *Lentilitoribacter* EG35 genome. The corresponding data are available on Zenodo under https://doi.org/10.5281/zenodo.13856673

The EG35 genome encodes the poly[(R)-3-hydroxyalkanoate] polymerase subunit *phaC* (EC 2.3.1.304) crucial for the biosynthesis of polyhydroxyalkanoates (PHAs), biodegradable polyesters that can be used for bioplastics production (17), and two other genes involved in PHA metabolism, the PHA synthesis regulator *phaR*, and the poly(3-hydroxybutyrate) depolymerase *phaZ* (EC 3.1.1.75). The strain’s potential to produce PHAs was confirmed *via* Nile Red staining (18). Strain EG35 harbors six BGCs coding for homoserine lactone signaling molecules, including one sharing 100% similarity to the BGC of kolossin, a peptide likely involved in interspecies communication (19). Moreover, four putatively novel BGCs coding for a terpene, arylpolyene, betalactone, and a ribosomally synthesized and post-translationally modified peptide are present.

## Data Availability

The genome sequence of *Lentilitoribacter* sp. strain EG35 has been deposited at DDBJ/ENA/GenBank under the BioProject PRJNA1135483 and the Whole Genome Shotgun accession number JBFNGQ000000000. The antiSMASH and Melange results of BGC, COG and Pfam annotations are available on Zenodo under https://doi.org/10.5281/zenodo.13856673. Further details can be found in Table 1.
